# Effect of Pirfenidone on TGF-β1-Induced Myofibroblast Differentiation and Extracellular Matrix Homeostasis of Human Orbital Fibroblasts in Graves’ Ophthalmopathy

**DOI:** 10.3390/biom11101424

**Published:** 2021-09-29

**Authors:** Shi-Bei Wu, Tzu-Yu Hou, Hui-Chuan Kau, Chieh-Chih Tsai

**Affiliations:** 1 Biomedical Commercialization Center, Taipei Medical University, Taipei 11031, Taiwan; barry0110@tmu.edu.tw; 2 Department of Ophthalmology, Kaohsiung Veterans General Hospital, Kaohsiung 813414, Taiwan; houtztz@gmail.com; 3 Department of Ophthalmology, Taipei Veterans General Hospital, Taipei 11217, Taiwan; hckau1234@yahoo.com; 4 School of Medicine, National Yang Ming University, Taipei 11221, Taiwan; 5 School of Medicine, National Yang Ming Chiao Tung University, Hsinchu 30010, Taiwan; 6 Department of Ophthalmology, Koo Foundation Sun Yat-Sen Cancer Center, Taipei 11259, Taiwan

**Keywords:** c-Jun N-terminal kinase, Graves’ ophthalmopathy, orbital fibroblast, p38, pirfenidone, transforming growth factor-β1

## Abstract

Pirfenidone is a pyridinone derivative that has been shown to inhibit fibrosis in animal models and in patients with idiopathic pulmonary fibrosis. Its effect on orbital fibroblasts remains poorly understood. We investigated the in vitro effect of pirfenidone in transforming growth factor-β1 (TGF-β1)-induced myofibroblast transdifferentiation and extracellular matrix (ECM) homeostasis in primary cultured orbital fibroblasts from patients with Graves’ ophthalmopathy (GO). The expression of fibrotic proteins, including α-smooth muscle actin (α-SMA), connective tissue growth factor (CTGF), fibronectin, and collagen type I, was determined by Western blots. The activities of matrix metalloproteinases (MMPs) and tissue inhibitors of metalloproteinases (TIMPs) responsible for the ECM homeostasis were examined. After pretreating the GO orbital fibroblasts with pirfenidone (250, 500, and 750 μg/mL, respectively) for one hour followed by TGF-β1 for another 24 h, the expression of α-SMA, CTGF, fibronectin, and collagen type I decreased in a dose-dependent manner. Pretreating the GO orbital fibroblasts with pirfenidone not only abolished TGF-β1-induced TIMP-1 expression but recovered the MMP-2/-9 activities. Notably, pirfenidone inhibited TGF-β1-induced phosphorylation of p38 and c-Jun N-terminal kinase (JNK), the critical mediators in the TGF-β1 pathways. These findings suggest that pirfenidone modulates TGF-β1-mediated myofibroblast differentiation and ECM homeostasis by attenuating downstream signaling of TGF-β1.

## 1. Introduction

Graves’ ophthalmopathy (GO) occurs in 25–50% of patients with Graves’ disease [1]. The pathological processes include inflammation of orbital connective tissues in the early course, followed by soft tissue expansion and fibrosis in the orbit, wherein orbital fibroblasts proliferate and differentiate into myofibroblasts, adipogenesis increases, and glycosaminoglycans (GAGs) accumulate in the extracellular matrix (ECM) [2,3,4]. In particular, fibrosis-associated morbidities, such as severe lid retraction, proptosis, limited ocular motility, and compressive optic neuropathy, are often poorly responsive to medical treatment and thereby require surgical intervention.

Transforming growth factor-β (TGF-β) has been implicated in various fibrotic disorders [5]. Prior studies have revealed a prominent TGF-β expression in the GO orbital fibroblasts [6,7]. Most importantly, TGF-β can induce tissue remodeling and fibrosis through myofibroblast transdifferentiation in GO orbital fibroblasts [8,9]. Our previous study showed that the TGF-β1-induced expression of connective tissue growth factor (CTGF), alpha-smooth muscle actin (α-SMA), fibronectin, tissue inhibitors of metalloproteinases (TIMP)-1, and TIMP-3 in GO orbital fibroblasts could be transduced by mitogen-activated protein kinase (MAPK) signaling pathways, in particular p38 and c-Jun N-terminal kinase (JNK) [10]. Myofibroblasts, the primary effector cells in fibrosis, can produce excessive amounts of ECM proteins and exhibit increased expression of α-SMA, CTGF, and fibronectin, leading to typical pathological features of GO.

Pirfenidone (5-methyl-1-phenyl-2[1*H*]-pyridone) is an oral anti-inflammatory and antifibrotic agent clinically indicated in the treatment of idiopathic pulmonary fibrosis [11]. In vitro and in vivo studies have confirmed its effect on the inhibition of myofibroblast differentiation, proliferation, and profibrotic cytokine release in lung, liver, heart, and renal tissues [12,13,14,15,16]. Throughout these years, pirfenidone has been used in several experimental disease models including renal and hepatic fibrosis [17], systemic sclerosis [18], wound healing [19], and ocular pathologies [20,21,22]. In terms of ocular disease, pirfenidone has been reported to play an antifibrotic and anti-inflammatory role in vitro, particularly in the orbital fibroblasts [20], pterygium fibroblasts [21], and retinal pigment epithelial cells [22]. However, its molecular targets and actions in GO have not been elucidated. In this in vitro study, we investigated whether pirfenidone was effective in the regulation of TGF-β1-induced myofibroblast transdifferentiation and ECM homeostasis in human GO orbital fibroblasts. Additionally, the action of pirfenidone on TGF-β1 transduction pathways underlying these processes was examined.

## 2. Materials and Methods

### 2.1. Primary Culture of Orbital Fibroblasts

The primary cultures of orbital fibroblasts were used in this study, as in our previous work [8]. All specimens were collected in accordance to the Declaration of Helsinki and with informed consent of the patients. The primary cultures of orbital fibroblasts were established from surgical intraconal specimens of four patients with GO (G1–G4) during orbital decompression surgery (one man and three women; mean age, 37.2 years). All GO patients received methimazole and achieved stable euthyroidism for at least six months before surgery, and had been precluded from radiotherapy and systemic corticosteroid treatment for at least one month before surgery. In addition, all four patients were in an inactive stage of GO and the disease activity was evaluated based on the modified clinical activity score (CAS) ranging between 1 and 2 [23]. Orbital fibroblasts were cultured in an incubator filled with an atmosphere of 5% CO_2_ at 37 °C, and the cultured medium contained 10% fetal bovine serum (FBS), penicillin G (100 U/mL), and streptomycin sulfate (100 μg/mL). All experiments were performed with cultured orbital fibroblasts between the 3rd and 5th passages.

### 2.2. Chemicals and Antibodies

Pirfenidone (#P2116) and the secondary antibodies against rabbit (#A5795) and mouse (#A9044) were acquired from Sigma-Aldrich (St. Louis, MO, USA). The human TGF-β1 recombinant protein (#P01137) and the mouse monoclonal antibodies against TIMP-1 (#MAB970) were acquired from R&D Systems, Inc. (Minneapolis, MN, USA). Antibodies including the rabbit polyclonal antibodies against CTGF (#ab6992), fibronectin (#ab2413), collagen type I (#ab34710), and α-SMA (#ab5694), the mouse monoclonal antibodies against p38 (#ab31828), p38 phosphorylation (p-p38, #ab4822), JNK (#ab208035), and JNK phosphorylation (p-p38, #ab76572) were all purchased from Abcam Inc. (Cambridge, UK).

### 2.3. Determination of Cell Viability

Cell viability was measured by a Trypan blue exclusion method, and was counted by using a hemocytometer. The number of viable cells was determined on the basis of their exclusion of 0.4% Trypan blue (Sigma-Aldrich). The relative cell viability was normalized by the value of cells without pirfenidone treatment and was expressed as mean ± SD of the results from three independent experiments.

### 2.4. Western Blot Analysis

An aliquot of 60–100 μg proteins was separated on 10% SDS-PAGE and blotted onto the PVDF membrane (Amersham-Pharmacia Biotech Inc., Buckinghamshire, UK). After blocking with 5% skim milk, the PVDF membrane was incubated with the primary antibody, followed by a horseradish peroxidase (HRP)-conjugated anti-rabbit or anti-mouse IgG antibody. The protein expression signals were detected by an enhanced chemiluminescence detection kit (Amersham-Pharmacia Biotech Inc.), according to the manufacturer’s instructions and were quantified by ImageScanner III with LabScan 6.0 software (GE Healthcare BioSciences Corp., Piscataway, NJ, USA).

### 2.5. MMP-2/-9 Enzyme Activity Assay

The MMP-2/-9 enzyme activities from the culture medium were measured by InnoZyme™ Gelatinase Activity Assay kit (Merck KGaA, Darmstadt, Germany) via a fluorogenic method [10]. According to the instructions, 100 μL of culture medium was diluted in activation buffer and incubated for three hours with thiopeptide substrate specific for MMP-2 and MMP-9. The released fluorescence of the cleaved substrate of MMP-2/-9 was monitored (ex: 320 nm; em: 405 nm). Triplicate tests were performed for each reaction in a 96-well plate, and the relative fluorescence unit ratios to control set were plotted.

### 2.6. Statistical Analysis

Analysis of Student’s *t* test and one-way ANOVA test were used by SPSS software and SigmaPlot (14.0 version). The statistical analysis was obtained from three independent experiments, respectively. Data are presented as means ± SD, and a *p* value of less than 0.05 and 0.01 was considered statistically significant.

## 3. Results

### 3.1. Pirfenidone Inhibited TGF-β1-Induced Fibrotic Protein Expression in GO Orbital Fibroblasts

**Figure 1:**
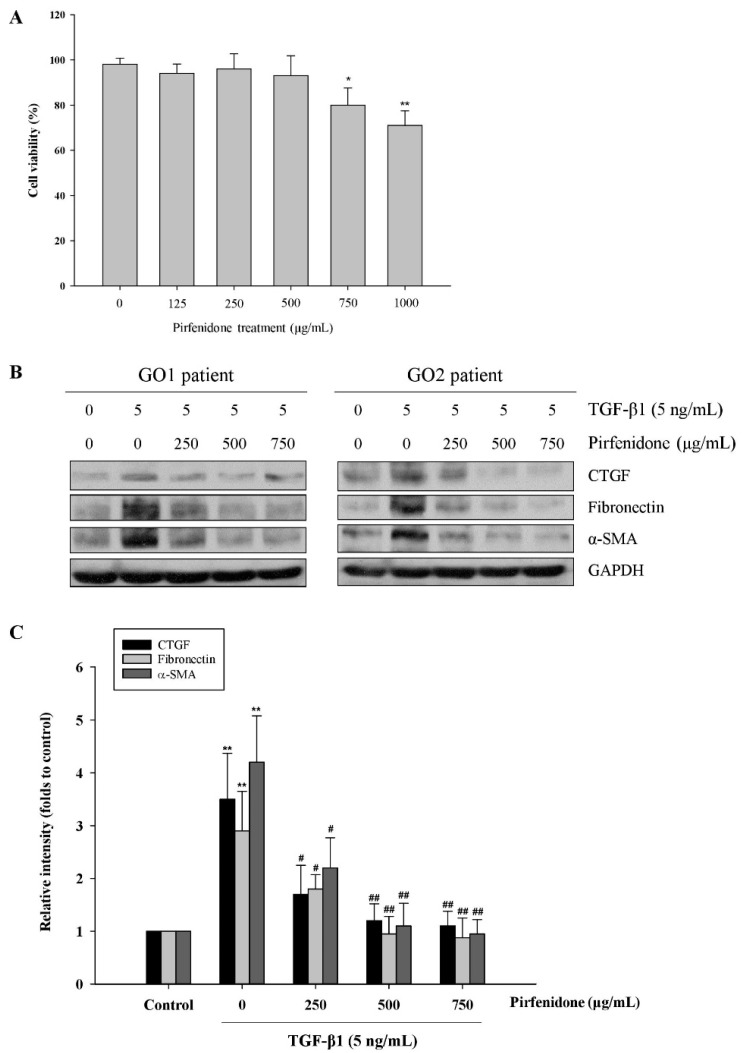
Inhibition of TGF-β1-enhanced fibrotic protein expression by pirfenidone in the primary cultures of human GO orbital fibroblasts. (**A**) After treatment of the GO orbital fibroblasts with various concentrations of pirfenidone (125, 250, 500, 750, and 1000 μg/mL, respectively) for 24 h, the cell viability was analyzed by a Trypan blue exclusion assay. (**B**) The GO orbital fibroblasts were pretreated with pirfenidone (250, 500 and 750 μg/mL, respectively) for one hour, followed by TGF-β1 (5 ng/mL) treatment for another 24 h. The protein expression levels of CTGF, fibronectin, and α-SMA were analyzed by Western blots. (**C**) The relative intensities of CTGF, fibronectin, and α-SMA expression normalized to each GAPDH were demonstrated. The relative intensities of the control without TGF-β1 treatment were defined as 1.0 and the other relative intensity (folds) is presented accordingly. The representative histogram is displayed based on the mean values of protein expression levels. Data are presented as means ± SD of the results from three independent experiments. * *p* < 0.05 and ** *p* < 0.01 vs. control without TGF-β1 treatment; # *p* < 0.05 and ## *p* < 0.01 vs. TGF-β1 treatment without pirfenidone.

**Figure 2:**
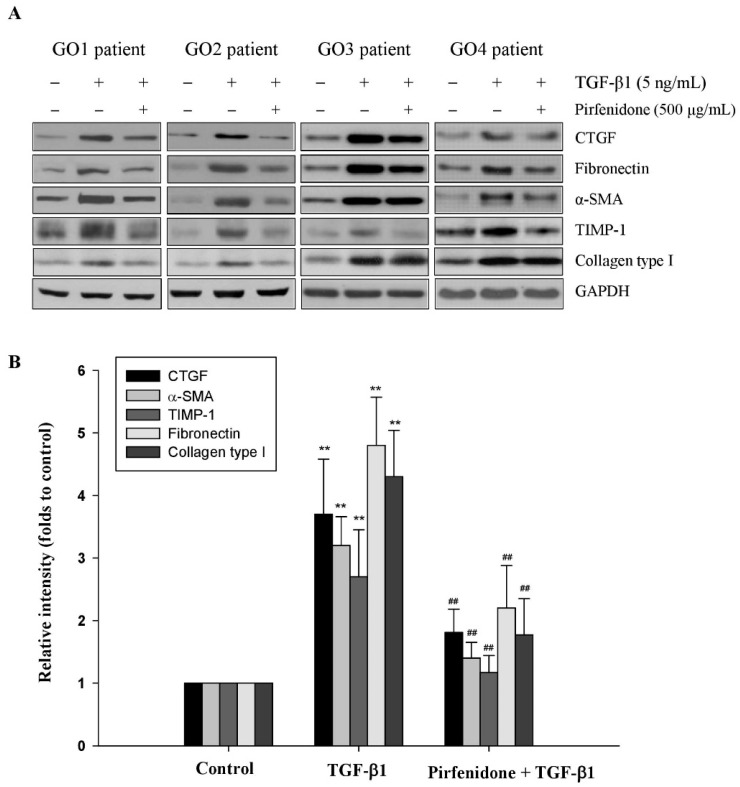
Regulation of TGF-β1-mediated matrix remodeling by pirfenidone in the primary cultures of human GO orbital fibroblasts. (**A**) The orbital fibroblasts from GO patients were pretreated with pirfenidone (500 μg/mL) for one hour, followed by TGF-β1 (5 ng/mL) treatment for another 24 h. The protein expression levels of CTGF, α-SMA, TIMP-1, fibronectin, and collagen type I were analyzed by Western blots. (**B**) The relative intensities of CTGF, α-SMA, TIMP-1, fibronectin, and collagen type I expression were normalized to each GAPDH. The relative intensities of the control without TGF-β1 treatment were defined as 1.0 and the other relative intensity (folds) is presented accordingly. By three independent Western blot experiments, together data from the same patient strain were averaged (GO1–GO4, *n* = 4). The representative histogram is displayed based on the mean values of protein expression levels. Data are presented as means ± SD of the results from three independent experiments. ** *p* < 0.01 vs. control without TGF-β1 treatment; ## *p* < 0.01 vs. TGF-β1 treatment without pirfenidone.

We have shown that TGF-β1 but not TGF-β2 could induce the expression of fibrotic proteins, including CTGF, fibronectin, and α-SMA, in the GO orbital fibroblasts [8]. In this study, we investigated the antifibrotic effect of pirfenidone on TGF-β1-treated orbital fibroblasts. First, we evaluated the toxicity of pirfenidone in the GO orbital fibroblasts to confirm the appropriate concentration by a Trypan blue exclusion assay. After treatment with various concentrations of pirfenidone (125, 250, 500, 750, and 1000 μg/mL) in the GO orbital fibroblasts for 24 h, the cell viability was significantly decreased at the concentration of 750 μg/mL (81.37% ± 7.54%, *p* = 0.0417) and 1000 μg/mL (71.84% ± 6.47%, *p* = 0.0018), respectively, as compared with the control without pirfenidone treatment (Figure 1A). Next, after treatment of the orbital fibroblasts with pirfenidone (250, 500, and 750 μg/mL, respectively) for one hour, followed by the addition of TGF-β1 (5 ng/mL) for another 24 h, the TGF-β1-induced upregulation of CTGF, fibronectin, and α-SMA was attenuated by pirfenidone in a dose-dependent manner (Figure 1B,C). Furthermore, the antifibrotic effect of pirfenidone was examined in another primary culture of orbital fibroblasts from four GO patients (GO1–GO4). We found that pretreatment of the orbital fibroblasts with pirfenidone (500 μg/mL) could significantly abolish 5 ng/mL TGF-β1-induced upregulation of CTGF, fibronectin, and α-SMA (Figure 2). These results confirmed the dose-dependent inhibition of fibrogenesis by pirfenidone in the TGF-β1-treated GO orbital fibroblasts.

### 3.2. Pirfenidone Diminished TGF-β1-Mediated ECM Metabolism in GO Orbital Fibroblasts

**Figure 3:**
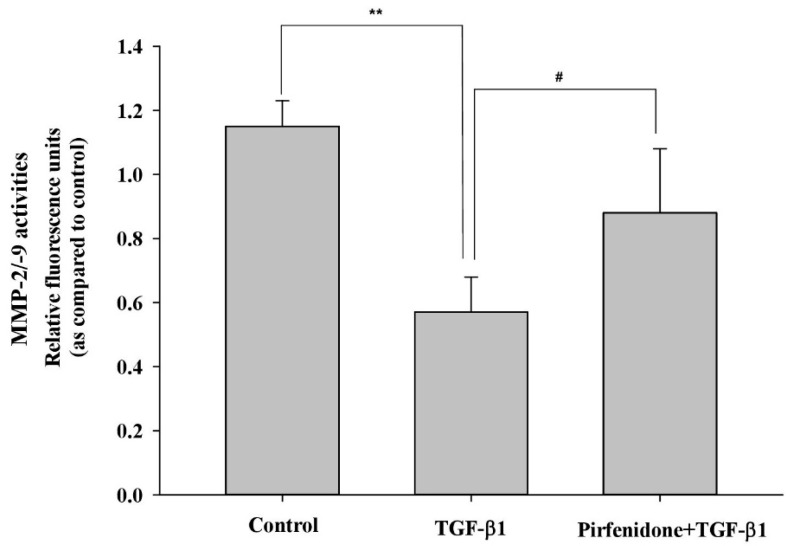
Recovery of the TGF-β1-inhibited MMP activities by pirfenidone in the primary cultures of human GO orbital fibroblasts. After pretreatment of the GO orbital fibroblasts with pirfenidone (500 μg/mL) for one hour, followed by the addition of TGF-β1 (5 ng/mL) for another 24 h, the enzyme activities of MMP-2/-9 from the cultured medium were determined. The representative histogram is displayed based on the mean values of relative fluorescence units. Data are presented as means ± SD of the results from three independent experiments. ** *p* < 0.01 vs. control without TGF-β1 treatment; # *p* < 0.05 vs. TGF-β1 treatment without pirfenidone.

The accumulation of ECM proteins associated with the remodeling of orbital tissues in GO, and collagen, a major matrix component, have been reported to be highly produced by activated orbital fibroblasts [23]. Thus, we first examined whether pirfenidone affected TGF-β1-mediated expression of collagen. The result indicated that pretreatment of the orbital fibroblasts with pirfenidone (500 μg/mL) could significantly abolish 5 ng/mL TGF-β1-induced upregulation of collagen type I (Figure 2). In addition, the turnover of ECM components is regulated by matrix-degrading proteolytic enzymes (i.e., MMPs) and their proteinase inhibitors (i.e., TIMPs). Since the accumulation of ECM proteins is associated with the remodeling of orbital tissues in GO, we examine whether pirfenidone affected TGF-β1-mediated TIMP and MMP activities. We found that pretreating the orbital fibroblasts with pirfenidone (500 μg/mL) could significantly abolish 5 ng/mL TGF-β1-induced TIMP-1 protein expression (Figure 2) and restore the MMP-2/-9 activities (Figure 3).

### 3.3. Pirfenidone Abolished TGF-β1-Induced p38 and JNK Phosphorylation in GO Orbital Fibroblasts

**Figure 4:**
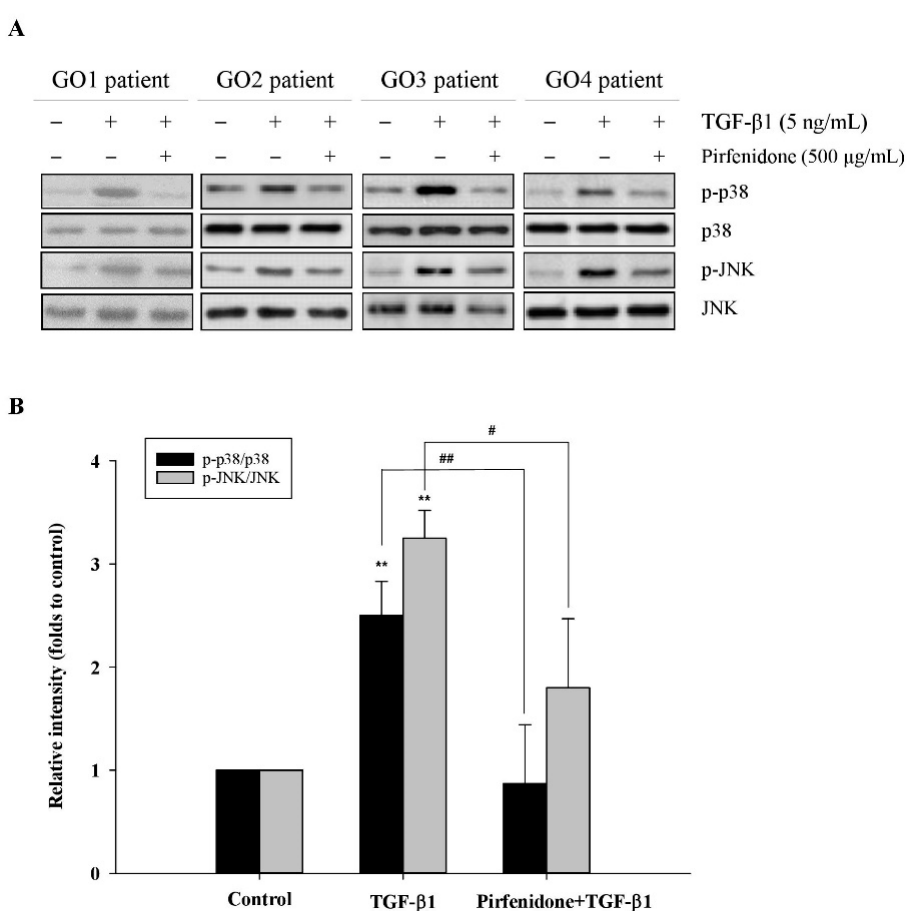
Abolishment of TGF-β1-induced p38 and JNK phosphorylation by pirfenidone in the primary cultures of human GO orbital fibroblasts. (**A**) After pretreatment of the GO orbital fibroblasts with pirfenidone (500 μg/mL) for one hour, followed by the addition of TGF-β1 (5 ng/mL) for another 24 h, p38 and JNK phosphorylation was determined by Western blots. (**B**) The expression ratios of p-p38 to p38 and p-JNK to JNK from the control without TGF-β1 treatment were defined as 1.0, and the other relative intensity (folds) is presented accordingly. By three independent Western blot experiments, data were averaged together from the same patient strain (GO1–GO4, *n* = 4). The representative histogram is displayed based on the mean values of protein expression levels. Data are presented as means ± SD of the results from three independent experiments. ** *p* < 0.01 vs. control without TGF-β1 treatment; # *p* < 0.05; ## *p* < 0.01 vs. TGF-β1 treatment without pirfenidone.

We have shown in our prior work that TGF-β1 induces the fibrotic process via the p38 and JNK signaling pathways in the GO orbital fibroblasts [10]. In this study, we examined whether pirfenidone inhibited the fibrotic process in the TGF-β1-treated orbital fibroblasts through the blockage of p38 or JNK phosphorylation. After treatment of the orbital fibroblasts with pirfenidone (500 μg/mL) for one hour, followed by the addition of TGF-β1 (5 ng/mL) for another 24 h, TGF-β1-induced p38 and JNK phosphorylation was inhibited (Figure 4). Thereby, p38 and JNK may be the targets of pirfenidone in TGF-β1-induced fibrogenesis of GO.

## 4. Discussion

Our prior research showed that TGF-β1 induced differentiation of orbital fibroblasts into myofibroblasts, which dominated the pathogenic processes of tissue remodeling and fibrosis in GO [8,10]. In this study, we observed that pirfenidone reduced TGF-β1-mediated myofibroblast differentiation and ECM homeostasis by inhibiting TGF-β1-induced expression of fibrotic proteins, fibronectin, collagenase type I, and TIMP-1. Furthermore, the TGF-β1-induced decrease in MMP-2/-9 activities could be prevented by pretreatment with pirfenidone. We supposed that the change of MMP-2/-9 activities by TGF-β1 may be regulated by overexpression of TIMP-1, and that pirfenidone treatment attenuated TGF-β1-induced p38 and JNK phosphorylation accompanied by a change of MMP-2/-9 activities in GO.

Pirfenidone is an anti-inflammatory and antifibrotic agent employed for the treatment of fibrotic processes in several animal models and the affected cells in the lung, liver, kidney, and heart [11,12,13,14,15,16]. Studies have confirmed the effect of pirfenidone on the inhibition of myofibroblast differentiation, fibrotic protein and profibrotic cytokine release, and ECM deposition [17,18,19,20,21,22]. Although it is evident that pirfenidone prevents fibrosis by reducing the reaction induced by TGF-β1, the definite signaling pathways remain to be determined. TGF-β1 propagates its signals by the Smad-dependent and Smad-independent, such as MAPK, i.e., p38, extracellular signal-regulated kinase (ERK), and JNK, phosphoinositide 3-kinase (PI3K), Wnt/β-catenin, and Akt (protein kinase B), pathways in fibrogenesis [24,25]. Targeting TGF-β1 downstream mediators may be a useful therapeutic strategy for fibrosis-related diseases. Conte et al. demonstrated that pirfenidone inhibited TGF-β1-mediated phosphorylation of Smad3, p38, and Akt in the primary human pulmonary fibroblasts [13]. Lv et al. found that pirfenidone could alleviate pulmonary fibrosis through regulating TGF-β1-mediated Smad2/3 phosphorylation and Wnt/glycogen synthase kinase-3β (GSK-3β)/β-catenin signaling pathways [26]. Several consecutive studies have confirmed the effect of pirfenidone on the TGF-β1/MAPK pathways in fibroblasts from different organs or tissues, including the epidura, pterygium, dermis, and lung [22,26,27,28,29,30,31]. Recently, we demonstrated that p38 and JNK could transduce TGF-β1-stimulated myofibroblast transdifferentiation in GO orbital fibroblasts [10]. TGF-β1-mediated p38 and JNK pathways independently contributed to the fibrotic process, and both p38 and JNK were essential for TGF-β1-mediated fibrosis in GO orbital fibroblasts. In this study, we revealed that pirfenidone directed against p38 and JNK phosphorylation to inhibit TGF-β1-induced fibrogenesis in GO orbital fibroblasts. In addition to our findings, pirfenidone showed greater potency than dexamethasone for the inhibition of IL-1β upregulated TIMP1 and collagen levels, as well as hyaluronic acid synthesis in GO orbital fibroblasts [20,32], at least in part through suppression of the MAPK-mediated pathways [20]. Both TGF-β1 and IL-1β are important proinflammatory cytokines that perpetuate orbital inflammation and subsequent tissue remodeling in GO. Based on these findings, it is reasonable to suggest a therapeutic potential of pirfenidone for the treatment of GO.

Tepezza (teprotumumab-trbw), a monoclonal antibody acting against insulin-like growth factor 1 (IGF-1), was recently approved by the United States Food and Drug Administration (FDA) for the treatment of active GO [33]. Patients with active GO treated with intravenous Tepezza resulted in better outcomes with respect to proptosis, clinical activity score, diplopia, and quality of life than those treated with placebo [33]. The advantage of pirfenidone over Tepezza includes the relatively lower price and wide application in clinical practice to treat idiopathic pulmonary fibrosis. We believe that the antifibrotic effect of pirfenidone would benefit the treatment and/or prevention of GO.

In conclusion, our results provided evidence that pirfenidone diminished the TGF-β1-induced fibrotic protein expression and restored the ECM homeostasis in the GO orbital fibroblasts. The antifibrotic effect of pirfenidone on the GO orbital fibroblasts may be attributed to the inhibition of the TGF-β1-induced JNK and p38 pathways.

## Data Availability

The data that support the findings of this study are available from the corresponding author upon reasonable request.
